# Supplementary material for: Bi-directional neuro-immune dysfunction after chronic experimental brain injury
Source: J Neuroinflammation. 2024 Apr 5;21:83. doi: 10.1186/s12974-024-03082-y (PMC10996305; doi:10.1186/s12974-024-03082-y)
Supplement: Supplementary file 1 — Supplementary Material 1 [file 12974_2024_3082_MOESM1_ESM.pdf]

Bi-directional neuro-immune dysfunction after chronic experimental brain injury

Rodney M. Ritzel[1,2]*, Yun Li[1], Yun Jiao[3], Sarah J. Doran[1], Niaz Khan[1], Rebecca J. Henry[1], Kavitha Brunner[1], David J. Loane[1], Alan I. Faden[1], Gregory L. Szeto[3], Junfang Wu[1]*

[1]Department of Anesthesiology and Shock, Trauma and Anesthesiology Research (STAR) Center, University of Maryland School of Medicine, Baltimore, MD, 21201 USA.

[2]Department of Neurology, McGovern Medical School, The University of Texas Health Science Center at Houston, TX, 77030, USA.

[3]Department of Chemical, Biochemical and Environmental Engineering, University of Maryland, Baltimore County, Baltimore, MD, 21250, USA.

*Corresponding authors

Rodney M. Ritzel, email: Rodney.M.Ritzel@uth.tmc.edu, phone: 713-500-5503

Junfang Wu, email: Junfang.Wu@som.umaryland.edu, phone: 410-706-5189

Supplementary Information

Supplemental Information includes Supplemental two figures and figure legends.

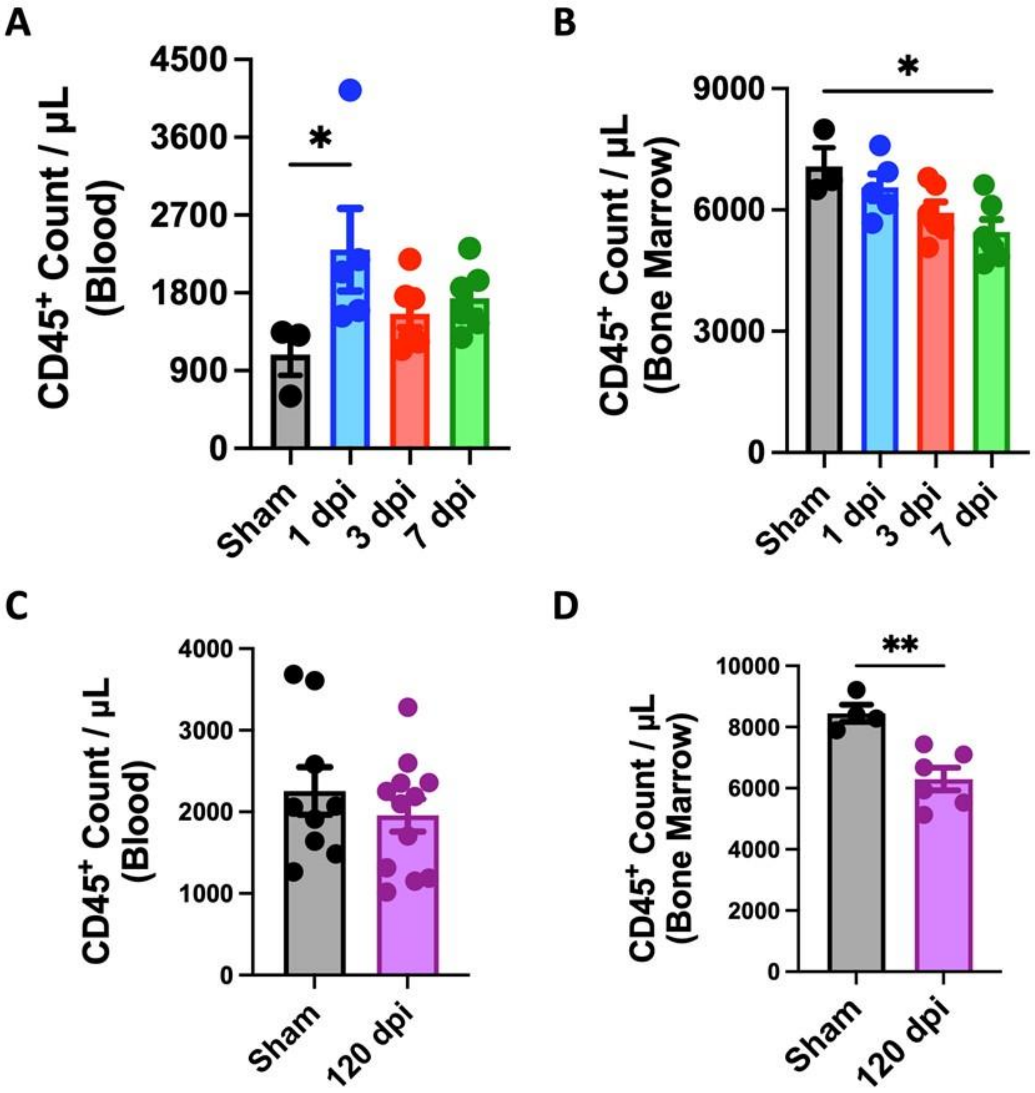

Supplementary Figure 1. Total leukocyte counts in the blood and bone marrow during the first week and after 120 days post-TBI. Flow cytometry assessment of CD45-positive leukocytes in the blood (**A**, **C**) and bone marrow (**B**, **D**) during acute (**A**-**B**) and chronic (**C**-**D**) timepoints after TBI. (**A**-**B**) n=3-6 mice/group, (**C**) n=9-12 mice/group, and (**D**) n=4-6 mice/group. Data were analyzed using one-way ANOVA group analysis with Tukey's test for multiple comparisons (**A**-**B**) or Student's T-test for two group comparisons (**C**-**D**). **p<0.01, *p<0.05.

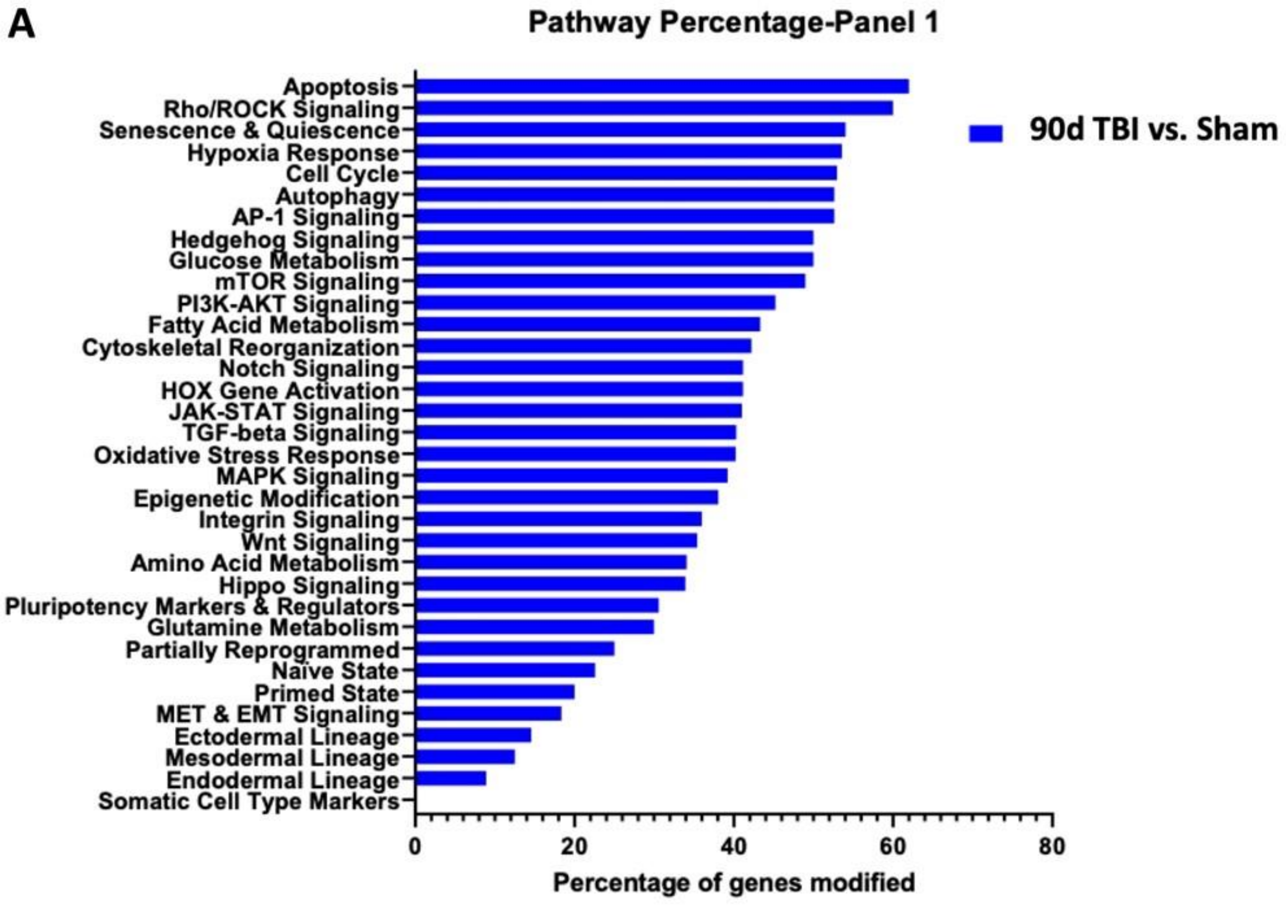

Supplementary Figure 2. Differentially regulated gene pathways in femoral bone marrow LSK+ cells after chronic TBI. (**A**) Pathway analysis based on gene annotations given by NanoString revealed high percentage of genes related to Apoptosis, Rho/ROCK signaling, Senescence & Quiescence, Hypoxia Response, and Cell Cycle pathways being modified by TBI (90 days post-injury). n=4-5 mice/group.
